# Autonomic responses to correct outcomes and interaction errors during single-switch scanning among children with severe spastic quadriplegic cerebral palsy

**DOI:** 10.1186/1743-0003-11-34

**Published:** 2014-03-08

**Authors:** Brian Leung, Tom Chau

**Affiliations:** 1 Institute of Biomaterials and Biomedical Engineering, University of Toronto, Rosebrugh Building, 164 College Street, Room 407, Toronto M5S 3G9, Canada; 2 Bloorview Research Institute, Holland Bloorview Kids Rehabilitation Hospital, 150 Kilgour Road, Toronto M4G 1R8, Canada

**Keywords:** Error processing, Autonomic response, Skin conductance, Heart rate, Access technology, Pediatric rehabilitation

## Abstract

**Background:**

The combination of single-switch access technology and scanning is the most promising means of augmentative and alternative communication for many children with severe physical disabilities. However, the physical impairment of the child and the technology’s limited ability to interpret the child’s intentions often lead to false positives and negatives (corresponding to accidental and missed selections, respectively) occurring at rates that frustrate the user and preclude functional communication. Multiple psychophysiological studies have associated cardiac deceleration and increased phasic electrodermal activity with self-realization of errors among able-bodied individuals. Thus, physiological measurements have potential utility at enhancing single-switch access, provided that such prototypical autonomic responses exist in persons with profound disabilities.

**Methods:**

The present case series investigated the autonomic responses of three pediatric single-switch users with severe spastic quadriplegic cerebral palsy, in the context of a single-switch letter matching activity. Each participant exhibited distinct autonomic responses to activity engagement.

**Results:**

Our analysis confirmed the presence of the autonomic response pattern of cardiac deceleration and increased phasic electrodermal activity following true positives, false positives and false negatives errors, but not subsequent to true negative outcomes.

**Conclusions:**

These findings suggest that there may be merit in complementing single-switch input with autonomic measurements to improve augmentative and alternative communications for pediatric access technology users.

## Background

### Access technologies

Persons with severe and/or multiple physical disabilities have limited or no means of interfacing with their immediate environment due to compromised motor functions and speech. Access technologies can restore communication and interaction for these individuals by training alternative physical and/or physiological pathways to express functional intent. A comprehensive review of access technologies can be found in Tai, Blain and Chau [1].

An access technology consists of an access pathway and a signal processing algorithm [1]. The access pathway is the input device that transduces a physical or physiological expression of function intent into an electrical signal. The signal processing algorithm processes the input signal into a corresponding output control signal. The output control signal is used to operate augmentative and alternative communication aids, environmental control units, or computers, thus enabling the access technology user to engage in function activity.

Access technologies have been developed for a variety of pathways. Strategically mounted pushbuttons, proximity sensors or microswitches can capture intentional gross physical movements such as head tilting [2] or tongue protrusions [3]. Extant fine motor control can be detected via electromyography [4,5], mechanomyography [6] and electrooculography [7]. Orofacial gestures can serve as access pathways when paired with computer vision [8-10] or facial thermography [11]. Applications of brain-computer interfaces as access technologies remain emergent, although many are still in research stages. A comprehensive set of brain imaging modalities, such as electroencephalography, near-infrared spectroscopy, transcranial Doppler, and fusion of modalities have been investigated for their potential as access pathways [12,13]. Autonomic nervous system signals can be trained and developed into access pathways [14]. These physiological pathways may be the only viable solution when the access technology user does not have any volitional physical control. The availability of multiple access pathway varieties maximizes the chances that prospective users can be outfitted with an access technology.

### Single-switch scanning

Single-switch access refers to access technologies whose output is a two-state ON/OFF signal. The output temporarily toggles to the ON state (i.e. switch activation) whenever the signal processing algorithm detects an expression of functional intent.

Non-trivial devices and software require their users to make selections from among multiple options. Thus, devices and software that support single-switch access must provide a protocol to facilitate one-of-*n* selection. To date, scanning remains the predominant protocol; the device or software automatically and sequentially visits each selectable item, one-by-one. A switch activation commits the presently selectable item as final. The speed of interaction is controlled by a configurable dwell time, which defines the amount of time each item becomes selectable [15].

Due to its inherent simplicity, single-switch scanning remains relevant despite the generally slow speed of interaction. Indeed, with currently available access technologies, many individuals with severe physical disabilities are only afforded one binary expression of functional intent (i.e. a YES/NO response). Single-switch scanning allows this solitary expression of intent to access a much wider array of possible outputs. Single-switch scanning has been deployed with many access technology users [15].

### Interaction errors

In the context of access technologies, we define an interaction error as a mismatch between user intent and the access technology output. In single-switch access, any interaction error is either a false positive or a false negative. A false positive is a switch activation without a deliberate expression of functional intent by the switch user. A false negative is switch inaction despite an expression of functional intent.

Interaction errors during single-switch scanning can be profoundly frustrating for the switch user. The user must wait for the desired choice to become selectable. Depending on the dwell time and the number of choices preceding the desired one, the wait time can range from several seconds to tens of seconds. Extending scanning to multiple dimensions (e.g. row-column scanning) or multiple levels (i.e. hierarchical scanning) can shorten, but not eliminate, the wait. Any false positive instance restarts scanning from the beginning regardless of the proximity to the desired choice at the time the inadvertent selection was made. Anytime a false negative occurs, the user must wait an entire scanning cycle before the desired choice becomes selectable again. Thus, interaction errors significantly slow down an already languid interaction modality. In particular, false positives can make it difficult to reach choices farther along the scanning sequence and so some choices may become inaccessible. Some switch users may see interaction errors as accentuating their inability to manipulate their environment, and this deprecating self-perspective can lead to a reinforced sense of learned helplessness and switch abandonment [16].

False positive and negative rates are functions of both user performance and the signal processing algorithm of the access technology. The ability to produce volitional, consistent and unambiguous expressions of functional intent can improve with user training. Enhanced signal processing algorithms may improve detection of more subtle expressions of functional intent and increase discriminative power against non-volitional ones. However, when user training is limited by the innate abilities of the individual and state-of-the-art algorithms cannot discern ambiguous expressions of functional intent any better, it might not be possible to reduce false positive and negative rates to levels that would enable functional communication.

### Physiological responses to interaction errors

The recognition of and response to reward and averse stimuli are fundamental, rational cognitive functions. These functions induce innate brain activity [17,18]. The stimulated brain regions may also be involved in autonomic regulation, and so the effects of the stimuli can propagate along efferent central and peripheral nervous pathways to influence heart rate, respiration and perspiration [19,20]. A large number of classical conditioning studies from psychophysiology literature have investigated heart rate, respiration characteristics, and electrodermal activity as tools to infer cognitive and affective mechanisms of the brain associated with the processing of reward and punishment [21-27].

The realization of an error elicits a response in the anterior cingulate cortex of the brain [18]. The anterior cingulate cortex is involved in rational cognitive functions such as decision-making, error detection, anticipation, attention and motivation. The cortical activity associated with error detection is characterized by a negative potential deflection in the electroencephalography recording of the anterior cingulate cortex [20]. This error-related negativity typically peaks at 50–150 ms after perception of the error. The peak amplitude tends to be greater when a person is motivated to perform a task favouring accuracy over speed [28] and when the person is certain that an error has occurred [20].

The anterior cingulate cortex plays a role in cardiovascular control [29] and activity in this cortex has been shown to modulate electrodermal activity [30]. It appears plausible that heart rate and electrodermal activity correlate with internal processing related to error detection. In the context of a modified Stroop task, Hajcak, McDonald and Simons [20] found, on average, cardiac deceleration following error detection and phasic skin conductance responses to errors having double the amplitude as compared to correct outcomes. These autonomic changes were observed in conjunction with error-related negative potentials. Similarly, Crone et al. [22] reported cardiac deceleration following penalty outcomes during the Iowa Gambling task and recovery to baseline levels following reward outcomes. They also observed greater skin conductance responses that scaled with the magnitude of the penalty. Furthermore, at least two other studies have reported the same autonomic response pattern to negative feedback among school-aged children and adolescents [31,32].

The possibility of detecting the switch user’s realizations of interaction errors will enable error handling strategies to circumvent false positives and false negatives. This can potentially improve the robustness of single-switch scanning even if false positive and negative rates cannot be reduced further. However, it is uncertain that the aforementioned autonomic nervous system responses can be found in persons with severe physical disabilities, because the brain lesions that compromise neuromotor functions can also affect the development of other brain functions [33].

The present case series investigated the autonomic responses of three pediatric single-switch users during engagement in a computer-based letter matching activity. Specifically, we addressed the questions of whether or not correct outcomes evoke specific autonomic response patterns and whether or not false positive and negative errors elicit cardiac deceleration and increased phasic electrodermal activity as reported in studies of neurotypical individuals.

## Methods

The methods presented here received ethics approvals from the research ethics boards of the Bloorview Research Institute (file number 10–106), the Toronto District School Board (file number 2009–2010–77) and the University of Toronto (protocol reference number 25295). All participants assented their participation and the parents consented their child’s participation in the study. The parents also consented to the reporting of details about their child in the participant profiles.

### Participants

Three male children participated in the present study under the pseudonyms Jason (nine years old), Charles (eleven years old) and Eric (seven years old). All three were affected by spastic quadriplegic cerebral palsy (Level Five on the Gross Motor Function Classification Scale [34]). They had no independent mobility and sat in manual wheelchairs while at school or in the community. At the time of the study, Jason was attending grade four in an integrated public elementary school where the majority of the student population was typically developing. Charles and Eric were attending grade six and grade two, respectively, at a segregated school where the student population was comprised entirely of children with mild to severe physical disabilities. Jason and Charles had intact vision, while Eric wore corrective glasses. All three participants had mild to severe hearing loss in one ear and usually wore a hearing aid to facilitate communication. Jason and Eric had limited speech, consisting mainly of single words that were intelligible only to familiar caregivers. Charles was considered non-verbal. He used a combination of a single vocalized sound and a rudimentary gesture to indicate yes and no. Nevertheless, all three participants demonstrated the ability to comprehend verbal instructions and react to verbal inquiries.

All three participants had experiences prior to the study with using computer applications as a regular part of their learning curriculum at school. Because of his younger age, Eric’s prior computer experience was limited to simple picture and word matching exercises with few selectable options. Jason and Charles worked on multiple choice language exercises such as word association and fill-in-the-blank writing activities. The requisite skills and abilities to use these single-switch educational software matched those necessary to complete single-switch experimental task of letter matching. The participants demonstrated that they were able to use these educational software with minimal help other than to physically implement a selection. They could perceive a set of options on the computer screen, independently track the states of scanning and volitionally make a selection. Also, all three participants had working knowledge of the English alphabet to complete the letter matching task.

Jason, Charles and Eric all used a vocal cord vibration switch [35] for single-switch access. The vocal cord vibration switch consisted of: 1) an accelerometer centred over the larynx and fastened by a thin elastic neck collar, and 2) a micro-controller-based processing unit that analyzed the accelerometry signal for periodicity associated with humming. Thus, the device could map inaudible and effortless hums to switch activations. All three participants were fitted with the switch at least six months prior to the study and had been casually and occasionally practicing with their switches at home and at school. Each participant had a history of unsuccessful switch-fittings prior to the vocal cord vibration switch. Past switch-fitting attempts involved wheelchair-mounted mechanical switches, non-contact switches by means of proximity and infrared sensors, and voice-activated switches. The vocal cord vibration switch was the access technology that each participant could most comfortably and consistently operate at the time of the study. Before the vocal cord vibration switch, the participants relied on caregiver-mediated single-switch access, where the limited role of the caregiver was to physically activate a switch on cue to each and every expression of functional intent by the participant. Thus, all three participants were familiar with the concepts of single-switch access and single-switch scanning.

### Instrumentation

**Figure 1:**
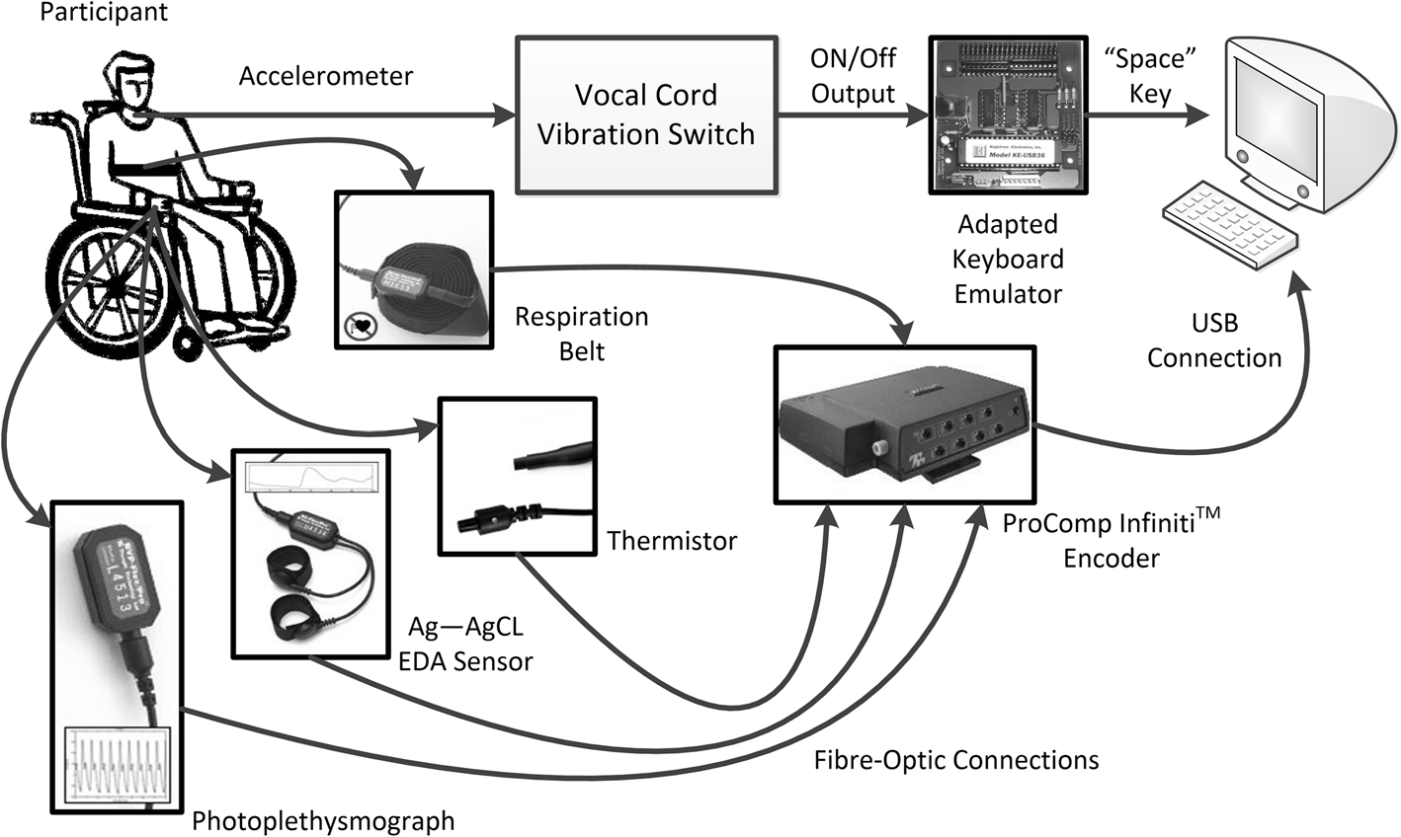
**Overview of the instrumentation.** An illustration of the instrumentation setup deployed for the three case studies. Images of the sensors, ProComp Infiniti encoder and the keyboard encoder were taken from the website of the the respective manufacturers.

Figure 1 summarizes the instrumentation used for the case studies. Each participant worked on a single-switch activity that was deployed on a desktop computer. The participant was positioned in front of the computer monitor at an appropriate distance and wheelchair tilt angle for comfortable viewing. The activity software was responsive to the keyboard’s “space” key as its single-switch control input. A Hagstrom (Lansing, New York) KE-USB36 hardware keyboard emulator was adapted to translate the single-switch output of the vocal cord vibration switch to a space bar signal to the computer, which the activity software interpreted as a selection. The experimenter also controlled the same desktop computer with a standard keyboard and mouse.

The single-switch computer activity software also collected physiological signals concurrently with each block of activity. Two 10 mm diameter Ag–AgCl electrodes emanating from a skin conductance sensor unit (Thought Technology SA9309M, Thought Technology Limited, Montreal, Canada) were attached to the medial phalanges of the index and middle fingers, in a bipolar configuration. A constant voltage of 0.5 V ran between the two electrodes during each recording. A single thermistor temperature sensor (Thought Technology SA9310M) was affixed to the anterior fingertip of the little finger. A photoplethysmography sensor (Thought Technology SA9308M) was fastened to the medial phalange of the ring finger for recording blood volume pulse waveforms. Jason wore the aforementioned sensors on his right hand because he typically wore a glove on his left hand to minimize injuries to his face from scratching during bouts of spasticity. Charles donned the sensors on his left hand because he was accustomed to having his left forearm strapped to his wheelchair tray for safety reasons. Eric chose to wear the sensors on his left hand. In addition, for each participant, a piezoelectric girth sensor (Thought Technology SA9311M) was secured thoracically over clothing but under the wheelchair restraints, in order to record respiratory waveforms.

The four physiological sensors were connected to the desktop computer via the Thought Technology ProComp Infiniti multi-modality encoder. The encoder sampled all four physiological signals at 256 Hz. The single-switch activity software annotated each set of physiological signals with events from the relevant block of letter matching activity.

### Single-switch activity

**Figure 2:**
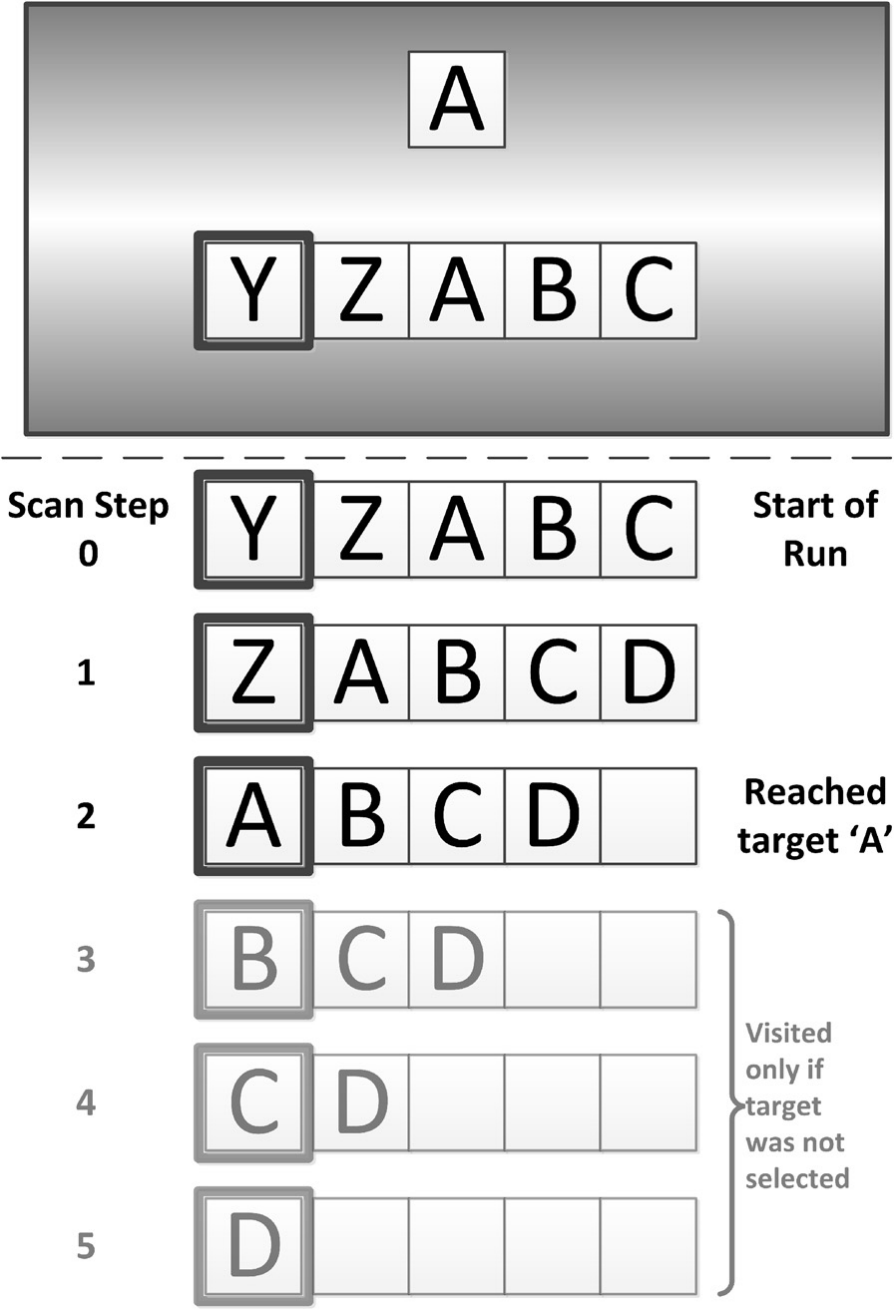
**The alphabet matching activity.** An illustration of the alphabet matching activity. In this example, the target letter is A and the first selectable choice letter is Y (i.e. the offset *x* equals to two). The graphic above the dash line illustrates the visuals that were shown to the participants on the computer screen. The graphic below the dash line demonstrates the complete set of letter buffer states for the current example.

The single-switch activity was a letter matching exercise as shown in Figure 2. The participant had to select the designated target letter when it became highlighted. Letter matching was chosen because all three participants were familiar with the alphabet and letters have a natural ordering.

One block of the activity consisted of matching 10 letters, one target letter at a time. As depicted in Figure 2, the target letter was shown at the top and a buffer of the next five selectable letters at the bottom. The buffer showed the next five letters that would become selectable. The number of choices remaining could be more or less than five letters. The leftmost letter of the buffer was always highlighted by a coloured border, denoting it as the selectable letter. To make the next choice letter selectable, the software would left-shift the choices by one position. The leftmost letter would leave the buffer. A new letter choice would move into the rightmost buffer position if there were more than five letter choices remaining. Otherwise, the rightmost position would become vacant. This particular visual presentation of scanning allowed the software to render the letters large enough for the participant to view, even if the target letter was more than five choices away. To help the participant perceive a change in choice letters, the left-shifting of the choice letters in the buffer was animated. These visual elements were designed to minimize visuo-spatial demands. The complexity of the overall visual presentation of the letter matching activity was comparable to the single-switch educational software that the participants were familiar with. The choices were always in alphabetical order so that the participant could anticipate the selection. Each newly selectable letter was enunciated by the computer. After a selection was made, one of two possible tones was presented depending on whether or not the selected and target letters matched. The dwell time was set at three seconds. This was the fastest dwell time that all three participants could manage for this task using the vocal cord vibration switch.

Target letters were drawn at random. The starting choice displayed in the buffer was the letter that preceded the target by *x* letters, where *x* was a random number between one and 13 inclusive. The target letter corresponded to an offset of zero and the letters wrapped from Z back to A. As an example, if the target letter is A and *x* is two, then the starting letter is Y (see Figure 2). Furthermore, three more letters followed the target letter on the list of letter choices. Thus, each iteration of letter matching could present up to a maximum of *x* + 4 unique choice letters. If no selection was made at all, then the current iteration would end after the last letter in the buffer was made selectable. In the previous example, the last letter is D and the iteration ends if a letter is chosen or after D is made selectable.

### Data collection

Each case study consisted of multiple one-hour data collection sessions at a rate of three to four sessions every two weeks. The sessions were conducted at the participants’ schools, during regular school hours. To avoid distractions participants were transferred from their classrooms to a nearby, designated data collection room. Each participant was accompanied by his educational assistant. Each assistant was highly familiar with the respective participant’s behaviours and responses.

At the beginning of each data collection block, there was a 30-second baseline period where the participants were instructed to remain calm and relax to the best of their abilities. In between blocks they rested for a minimum of two minutes. The session goal was to have the participants complete four to six blocks. In practice, there were sessions with as few as three blocks, when the participants were in some discomfort due to muscle spasticity and required longer breaks. At the other extreme, some sessions entailed as many as seven blocks due to participant non-compliance, which necessitated the repetition of certain blocks within the available session time.

For each block, the alphabet matching software collected the aforementioned physiological signals and a full history of each letter to be matched, including the target letter, a list of letter choices that were selectable up to the moment of switch activation, the selected letter if a selection was made, and all detected switch activations. The data were recorded as time-stamped entries in computer log files. The experimenter documented the participants’ behaviours and noted whether or not each switch activation was deliberate.

### Data analysis

#### Objective performance measures of single-switch use

The objective performances of the participants in using their vocal cord vibration switch were quantified in terms of sensitivity and specificity to intentional hums. Values for these two metrics were calculated from the counts of true positive instances *n*_TP_, false negative instances *n*_FN_, true negative instances *n*_TN_ and false positive instances *n*_FP_. Sensitivity measured the vocal cord vibration switch’s responsiveness to intentional hums while specificity measured how often the switch activations corresponded to intentional hums. The counts of *n*_TP_, *n*_FN_, *n*_TN_ and *n*_FP_ were obtained from the computer log files but were also validated against the experimenter’s notes. In particular, intentional switch activations on non-target letters, which the software counted as false positives because of the non-target selections, were corrected to true positives due to the expressions of functional intent. Similarly, unintentional switch activations on target letters were counted as false positives rather than true positives as according to the software. These corrections were only implemented to a small number of false positive and true positive instances and, in particular, corrections from false positive to true positive were only needed to accommodate the participants’ occasionally deviant behaviour from the task protocol.

#### Pre-processing of the physiological signals

The goal of pre-processing the skin conductance signals was to extract the phasic skin conductance response and tonic skin conductance level components. The skin conductance signals were especially prone to spike noises and small gaps of near-zero values due to poor sensor-to-skin contacts. Median filtering (window size of 513 samples at sampling rate of 256 Hz) was used to attenuate the effects of these noise sources. Skin conductance signals were also smoothed using locally weighted scatterplot smoothing (LOWESS) [36] with a span of three seconds in order to attenuate nominal variations of the Ag–AgCl sensor measurements and repair gaps in measurements.

Visual inspection of the treated skin conductance signals revealed that the standard trough-to-peak amplitude analysis [37] could not adequately isolate non-specific and event related skin conductance responses. There were numerous instances of overlapping phasic skin conductance responses and so the standard method would underestimate the amount of phasic electrodermal activity [38]. More recently, signal deconvolution methods have been presented as being more sensitive to changes in phasic activity even in the presence of overlapping responses [37]. The skin conductance signal measures total changes in electrical conductance along a path between the two Ag–AgCl electrodes, and increases in activity of the eccrine sweat glands along that path generally lead to increases in conductance. Eccrine sweat gland activity is modulated by the sympathetic nervous system, which controls the secretion of ionic fluid into the sweat ducts. The fluctuations in conductance due to a sweat gland’s response to a unit innervation can be interpreted, from a signals and systems perspective, as an impulse response of the sweat glands. The skin conductance signal thus can be considered as the convolution of an impulse response function and a driving signal that represents the pattern of sympathetic nervous system innervations. Therefore, the premise of signal deconvolution is to recover the driving signal from the skin conductance signal using an appropriate impulse response function. The algorithms differ in their treatments of deconvolution artifacts, the types of recovered driving signals, and the measures of tonic and phasic electrodermal activities [38-40].

The continuous decomposition analysis by Benedek and Kaernbach [38], which was chosen for the present analysis, assumes the skin conductance signal to be the convolution of a single impulse response function by the sum of the tonic and phasic driving signals. The impulse response function has bi-exponential form

(1) $$f(t)=g\times (e^{\frac{-t}{\tau_{1}}}-e^{\frac{-t}{\tau_{2}}})$$

with time constant *τ*_1_≤*τ*_2_ and models sweat gland secretions entering into and then dissipating from the sweat duct. For each signal, the algorithm finds parameters for the impulse response function that maximize the number of distinct and isolated peaks while minimizing the amount of negativity in the phasic driving signal. Negativity in the driving signal, which cannot be explained by the aforementioned physiological framework, is an artifact of assuming a fixed impulse response function. This algorithm has the advantage of producing a single continuous measure of skin conductance response, the phasic driving signal, which could simplify subsequent analyses of phasic electrodermal activity [38]. A normalized measure of phasic electrodermal activity over a time window can be obtained simply by computing the area under the phasic driving signal within the time window and then dividing the area by the time window duration. This normalized measure is reported in units of micro-Siemens.

Blood volume pulse signals were used to derive instantaneous heart rate values in order to investigate changes in cardiovascular activity during single-switch scanning. To attenuate sensor and spike noise due to motion artifacts and poor sensor-to-skin contacts, each blood volume pulse signal was treated with median filtering (window size of 17 samples at sampling rate of 256 Hz) followed by wavelet de-noising (five level symlet 8 decomposition and soft thresholding using heuristic Stein’s Unbiased Risk Estimate). Then, the signals were band-pass filtered using a Chebyshev Type 2 kernel, retaining only frequency content corresponding to 60–200 beats per minute, which encompassed the range of possible heart rates for children with cerebral palsy [41]. The band-passed signals were forwarded to a peak detection algorithm in order to extract inter-beat interval values and, thus, instantaneous heart rate values. To further attenuate effects of noise, each time series of instantaneous heart rate values were smoothed with a 7^*th*^ order moving average filter, relying on the assumption that heart rate can be locally stationary around a small neighbourhood of beats.

Similarly, instantaneous respiration rates were calculated from the respiration waveforms. The pre-processing of the respiration waveforms mirrored that for blood volume pulse signals. The parameters of the time domain filters were adjusted for the slower-varying respiration waveform. Specially, the median filter had a window size of 65 samples. The band-pass filter had passband frequencies that corresponded to 10–90 breaths per minute. Each times series of instantaneous respiration values were smoothed with a 3^*rd*^ order moving average filter.

The fingertip temperature signals were least susceptible to measurement noise. Pre-processing of the temperature signals consisted only of LOWESS smoothing [36] to attenuate nominal fluctuations of the thermistor measurements. A neighbourhood of samples spanning one second was used for the local regression to determined the corresponding smoothed signal value.

Each signal was manually inspected after pre-processing. Signals that remained noisy were omitted from further analysis. In total, 18 out of the 359 blood volume pulse signals, 41 out of the 328 respiration waveforms and 18 out of the 348 temperature signals were omitted. The skin conductance signals were manually inspected prior to decomposition into tonic and phasic components, and 45 out of the 359 skin conductance signals were omitted.

#### Statistical analysis

The present analysis examined changes in feature values of the pre-processed physiological signals due to events of interest during the single-switch activity. The types of events of interest included: 

1. Presentation of the first target letter. This event represented the transition from non-activity (baseline) to activity engagement.

2. Switch activation corresponding to a true positive instance.

3. Switch activation corresponding to a false positive instance.

4. A choice letter, corresponding to a true negative instance, at the moment it became selectable.

5. A choice letter, corresponding to a false negative instance, at 1.5 seconds after it became selectable. This delay corresponded to half of the dwell time and accounted for the time uncertainty in which the participant attempted the unsuccessful switch activation(s).

Suppose an event of interest occurred at time *t*_0_. Let us define the time interval [ *t*_*p*_,*t*_0_) with *t*_*p*_≤*t*_0_ as the analysis time window prior to the event and the time interval (*t*_0_,*t*_*a*_] with *t*_0_≤*t*_*a*_ as the analysis window after the event. The feature values *f*_*p*_ and *f*_*a*_ were computed from the physiological signal of interest during the time interval [ *t*_*p*_,*t*_0_) and (*t*_0_,*t*_*a*_], respectively. The difference in feature values *d*_*f*_ was calculated as *d*_*f*_=*f*_*a*_-*f*_*p*_. The difference values for the same feature and same type of event of interest were pooled together by participants. The Wilcoxon signed-rank test [42] for zero population median at 5% significance level was applied on each set of pooled difference values. Rejection of the null hypothesis would suggest an overall difference of the feature in consideration before and after the event of interest.

The features that were considered in the present analysis included the mean heart rate, the mean respiration rate, the normalized measure of the phasic skin conductance response, the mean tonic skin conductance level, and the mean temperature.

For the first event type, the analysis time window parameters *t*_*p*_ and *t*_*a*_ were set such that [ *t*_*p*_,*t*_0_) spanned the entire non-activity section and (*t*_0_,*t*_*a*_] spanned the entire activity engagement section. For the other four event types, the prior and after analysis time windows were balanced (i.e. *t*_*p*_=*t*_*a*_) and had possible durations of three, five and seven seconds. The different durations were considered because of differences in the response times of the physiological signals. Durations of more than seven seconds were not considered because the corresponding prior and after time windows would span more than two choice letters (at a scanning dwell time of three seconds) and the association of the feature values to an event of interest would become ambiguous.

## Results

### Performance with the vocal cord vibration switch

Table 1 summarizes the participants’ performance with the vocal cord vibration switch. All three participants completed comparable numbers of study sessions and numbers of single-switch activity blocks. Eric completed more sessions and blocks because of non-compliance during some of his earlier sessions.

**Table 1:** A summary of the three participants’ involvement in their case studies and their performance with the vocal cord vibration switches

**Participant**	***n*** **_Session_**	***n*** **_Block_**	***n*** **_TP_**	***n*** **_TN_**	***n*** **_FP_**	***n*** **_FN_**	**Sensitivity (%)**	**Specificity (%)**
							**[Mean ± SD]**	**[Mean ± SD]**
Charles	22	123	830	3471	237	75	92.76±9.97	92.45±7.19
Jason	22	107	666	3591	288	103	83.98±21.97	91.60±8.21
Eric	29	144	902	4477	376	208	82.99±18.13	90.18±10.35

The *n*_Session_ and *n*_Block_ columns report the total numbers of study sessions and activity blocks that each participant completed. The *n*_TP_, *n*_TN_, *n*_FP_ and *n*_FN_ columns show the counts of true positive, true negative, false positive and false negative instances, respectively. The abbreviation “SD” refers to standard deviation.

The sensitivity and specificity values in Table 1 are average values calculated from the activity block–level sensitivities and specificities achieved by each respective participant. All three participants used the vocal cord vibration switch proficiently, which was characterized by average sensitivities exceeding 80% and specificities greater than 90%. The relatively large standard deviations associated with the sensitivity and specificity values indicated that the access solution was perfect sometimes but ineffective at other times. These fluctuations in performance could be attributed, in part, to session-to-session variation in accelerometer placement over the larynx and the participants’ fatigue and attention levels.

### Behavioural observations

There were noticeable differences in the physical states of the three participants during single-switch activity. Charles and Eric were generally relaxed unless they were bothered by bouts of muscle spasticity. In contrast, Jason’s whole body became mild to moderately tense throughout the letter matching activity. This led to visible thermoregulatory sweating and the occasional laboured switch activation attempt.

All three participants reacted physically to interaction errors. A strong vocalization and gross movements of the arms and head followed most false positive instances. They appeared to be genuinely surprised and annoyed by false positive errors. With false negatives, the participants often would immediately attempt another switch activation, by way of a louder second hum, even if the dwell time was about to elapse. Unfortunately, these stronger vocalizations sometimes exhibited diminished periodicity and were thus not detected as intentional switch activations [35,43,44]. These effortful vocalizations may have altered periodicity characteristics of the measured vibratory signal via the increase in expiratory muscle tension that can accompany vocalizations of increasing loudness [45] or the introduction of higher pitches, which are known to heighten the excursion of the larynx and hyoid in the humming [46].

Participant compliance was not a problem in any of the case studies. Each participant understood the letter matching activity and the experiment protocol. Occasionally, Charles and Eric would select letters of their choice instead of the designated target letters. Their educational assistants remarked that these deviant behaviours could be attributed to playfulness as opposed to boredom with the activity.

All participants favoured accuracy over speed of task performance. All of them knew that because the speed of single-switch scanning was fixed by the dwell time, performing the letter matching accurately was the best way to showcase their skills with the vocal cord vibration switch. They were motivated to achieve a perfect score (i.e. matching all the target letters correctly) for each activity block, despite having been told that the score was irrelevant to the objectives of the case studies. Jason, in particular, asked for his score at the end of most activity blocks. However, on multiple occasions the participants ended an activity block prematurely, by deliberately selecting the first letter of each new sequence of choices, when high task accuracy (e.g. 8 out of 10 correct matches or better) was no longer attainable. This behaviour is consistent with the learned helplessness observed in single-switch users with profound and multiple disabilities [16].

### Autonomic responses to single-switch activity events

Table 2 summarizes the statistical analyses of the autonomic responses of Jason, Charles and Eric, respectively, during single-switch activity.

**Table 2:** The autonomic responses of the three participants during single-switch activity

**Participant**	**Event**	***t***_***p***_	***t***_***a***_	***d*** **_HR_**	***d*** **_RR_**	***d*** **_SCR_**	***d*** **_SCL_**	***d*** **_TMP_**
Jason	AE	–	–	**5.65±10.02**	**4.10±5.01**	-0.01±0.36	**1.22±1.87**	**-0.329±0.655**
		3	3	-0.25±15.85	**-0.31±5.94**	**0.34±0.94**	-0.06±0.66	**-0.023±0.057**
	TP	5	5	-0.88±16.27	0.04±7.20	**0.23±1.15**	-0.07±0.92	**-0.039±0.093**
		7	7	-1.18±15.74	0.21±7.57	**0.17±1.29**	**-0.05±1.11**	**-0.055±0.127**
		3	3	0.33±16.31	0.21±6.45	**-0.03±1.12**	**0.04±0.66**	**-0.026±0.056**
	TN	5	5	0.34±17.34	0.33±7.67	**-0.06±1.23**	**0.07±0.89**	**-0.042±0.091**
		7	7	0.37±17.07	0.29±7.92	**-0.08±1.26**	**0.10±0.98**	**-0.060±0.123**
		3	3	**-1.99±16.72**	**-0.42±6.24**	**0.25±1.12**	**-0.05±0.75**	**-0.020±0.050**
	FP	5	5	-1.76±17.79	-0.49±7.20	0.14±1.01	**-0.02±0.87**	**-0.033±0.080**
		7	7	-1.18±17.50	-0.35±7.73	0.04±0.69	**0.07±0.61**	**-0.049±0.107**
		3	3	0.65±21.32	0.15±6.94	-0.04±1.61	**0.13±1.34**	**-0.035±0.053**
	FN	5	5	2.18±22.85	-0.43±8.50	-0.21±2.22	0.14±1.99	**-0.059±0.085**
		7	7	2.99±21.56	-0.94±8.86	**-0.49±1.63**	**0.38±1.29**	**-0.082±0.117**
Charles	AE	–	–	-1.35±8.39	0.70±4.38	**0.15±0.26**	**0.39±0.75**	0.071±0.353
		3	3	-0.04±13.82	0.16±6.06	**0.10±0.66**	**0.01±0.22**	**-0.005±0.036**
	TP	5	5	-0.09±15.37	0.31±6.69	**0.08±0.54**	**0.03±0.31**	**-0.008±0.058**
		7	7	0.04±15.48	0.42±6.83	**0.06±0.52**	**0.07±0.32**	**-0.012±0.079**
		3	3	0.04±13.11	**-0.04±5.24**	**-0.01±0.57**	**0.02±0.17**	**-0.007±0.038**
	TN	5	5	0.09±14.37	**-0.11±6.18**	**-0.01±0.49**	**0.03±0.23**	**-0.011±0.060**
		7	7	0.08±14.13	-0.12±6.52	0.00±0.44	**0.04±0.27**	**-0.015±0.081**
		3	3	**-1.96±13.24**	**1.06±5.55**	**0.27±0.76**	**0.04±0.20**	**-0.007±0.043**
	FP	5	5	**-2.52±14.38**	0.84±6.73	**0.16±0.56**	**0.08±0.27**	**-0.012±0.068**
		7	7	**-2.50±13.92**	0.72±6.95	**0.10±0.54**	**0.11±0.35**	**-0.017±0.092**
		3	3	-1.22±17.54	1.06±4.92	**0.15±0.32**	**0.02±0.06**	0.001±0.032
	FN	5	5	-1.72±19.44	0.96±5.98	0.12±0.34	**0.04±0.09**	-0.000±0.052
		7	7	-1.11±19.44	1.02±6.15	0.10±0.35	**0.05±0.13**	-0.004±0.071
Eric	AE	–	–	**-5.45±9.05**	0.41±3.50	**0.02±0.11**	**0.19±0.29**	**0.035±0.376**
		3	3	-1.05±13.31	**0.68±4.29**	**0.07±0.40**	**0.01±0.11**	0.000±0.031
	TP	5	5	-0.78±14.58	**0.86±5.02**	**0.05±0.33**	**0.01±0.17**	0.000±0.048
		7	7	-0.69±14.26	**0.72±5.18**	**0.04±0.29**	**0.03±0.20**	-0.000±0.064
		3	3	0.14±13.49	**-0.07±3.95**	**-0.02±0.43**	**0.01±0.15**	-0.001±0.030
	TN	5	5	0.19±14.76	**-0.09±4.74**	**-0.02±0.36**	**0.02±0.22**	-0.002±0.047
		7	7	0.13±14.50	**-0.09±5.03**	**-0.02±0.34**	**0.03±0.27**	-0.002±0.062
		3	3	-1.21±14.68	**0.80±5.03**	**0.20±0.61**	**0.03±0.19**	-0.001±0.032
	FP	5	5	**-1.80±16.16**	**0.83±5.95**	**0.17±0.50**	**0.04±0.31**	-0.001±0.049
		7	7	**-1.82±14.60**	**0.68±6.24**	**0.12±0.41**	**0.06±0.28**	-0.000±0.065
		3	3	-1.15±15.24	**1.84±5.93**	**0.12±0.38**	**0.02±0.09**	0.004±0.024
	FN	5	5	-2.46±16.66	**2.01±6.29**	**0.13±0.28**	**0.04±0.14**	**0.007±0.038**
		7	7	-2.99±16.06	**2.15±6.41**	**0.12±0.26**	**0.06±0.20**	0.009±0.052

The event abbreviations “AE”, “TP”, “TN”, “FP” and “FN” refer to activity engagement, true positive, true negative, false positive and false negative respectively. The ***t***_***p***_ and ***t***_***a***_ columns show the lengths of the analysis windows prior to and after each event (except for the “AE” event where the window lengths were variable). The ***d***_***HR***_***,d***_***RR***_***,d***_SCR_***,d***_***SCL***_ and ***d***_***TMP***_ columns report the mean **±** standard deviation of the pooled difference values for heart rate (beats per minute), respiration rate (breaths per minute), phasic skin conductance response (micro-Siemens), tonic skin conductance level (micro-Siemens) and temperature (degrees Celsius), respectively. A bolded entry indicates rejection of the zero-median null hypothesis (Wilcoxon signed-rank test, ***p<0.05***).

To validate the application of the continuous decomposition analysis for the skin conductance signals, we compare the optimized values for the time constants *τ*_1_ and *τ*_2_ of the bi-exponential impulse response function against reference values reported by Benedek and Kaernbach [38]. The average optimized values for *τ*_1_ for Jason, Charles and Eric were 1.22±0.42 seconds, 1.32±0.56 seconds and 1.33±0.39 seconds respectively, which were comparable to the average value of 0.96±0.56 seconds reported by Benedek and Kaernbach. The average optimized values for *τ*_2_ for Jason, Charles and Eric were 3.86±1.28 seconds, 4.19±1.94 seconds and 4.24±1.75 seconds respectively, which once again were similar to the reference average value of 3.76±1.88 seconds.

All three participants showed non-specific temperature responses to correct outcomes and interaction errors. The magnitude and direction of temperature change were almost the same across the true positive, true negative, false positive and false negative events. Also, tonic skin conductance level was not particularly labile to the events of single-switch activity. This was not unexpected because event stimuli usually evoke phasic, rather than tonic, electrodermal activity [21].

#### Activity engagement

Jason’s autonomic changes from baseline to activity engagement were characterized by increases in heart rate, respiration rate and tonic skin conductance level, and a decrease in temperature. These changes could be attributed to widespread muscle tightness during many of the activity blocks. The visible sweating resulted in elevated skin conductance level. The cooling effect of thermoregulatory sweating might have resulted in an average drop in temperature.

Charles and Eric autonomic changes were characterized by a decrease in heart rate and mild increases in skin conductance response, skin conductance level and temperature. It was not surprising that their autonomic changes were alike because both participants were similarly relaxed during baseline and activity engagement. The increased electrodermal activity likely reflected cognitive processing during letter matching. The decrease in heart rate and increase in respiration rate could have stemmed from focused attention to letter matching upon activity engagement [47].

#### True positive

All three participants responded on average to true positive outcomes with elevated phasic electrodermal activity. Jason’s average skin conductance response to true positives was greater than his skin conductance responses to interaction errors, which is opposite to the findings of the study by Hajcak, McDonald and Simons [20].

In addition, Jason and Eric responded with a decrease in heart rate while Charles and Eric responded with an increase in respiration rate. The mild increase in respiration rate could be due to a quick recovery breath following a hum.

#### True negative

The autonomic changes associated with true negative instances were minimal for all three participants. The average difference values of the heart rate, respiration rate and skin conductance response deviated farther from zero for Jason because of his elevated body tone during letter matching. The minimal autonomic responses were expected because true negative events consisted mostly of idle transitions from one non-target choice letter to another, during which the participants’ underlying cognitive and affective states should remain unchanged.

#### False positive

All three participants responded on average to false positive errors with cardiac deceleration and elevated phasic electrodermal activity. For Charles and Eric, these increases in phasic electrodermal activity nearly doubled the changes associated with true positive outcomes. These findings echo those reported by Hajcak, McDonald and Simons [20], by Crone et al. [22,31] and by Groen et al. [32].

The respiration rate on average increased for Charles and Eric and decreased for Jason following false positives. This difference in response could, in part, be attributed to individualized physical reactions to errors. Jason’s body tone tended to increase further which briefly interfered with breathing due to prolonged apnea. Charles and Eric tended to utter a quick vocalization which cut short the current breathing period.

#### False negative

Charles and Eric responded on average to false negative errors with cardiac deceleration and elevated phasic electrodermal activity. These results also confirm findings from literature. However, the increases in phasic electrodermal activity were more modest than the elevations in response to false positive errors. The respiration rate increased for the same reason as false positive errors. For Eric, the exaggerated increases in respiration rate associated with false negatives could be due to his tendency to reattempt switch activation multiple times.

Jason’s autonomic responses to false negatives were markedly different; they were characterized by increased heart rate, decreased respiration rate, decreased phasic electrodermal activity and increased tonic electrodermal activity. Jason’s efforts at reattempting switch activation could provide an explanation for this response pattern. As with false positive errors, Jason reacted to false negatives with increased body tone. The subsequent attempts at humming required more physical effort, which in turn might have resulted in a further increase in heart rate. The combination of increased body tone and repeated attempts at humming resulted in prolonged apnea. The increased physical activity may have induced thermoregulatory sweating [48], saturating the sweat glands with ionic fluid and thereby increasing tonic electrodermal levels [40].

## Discussion

### Autonomic responses to single-switch activity events

The results of the physiological signal analyses revealed distinct autonomic response patterns to activity engagement, correct outcomes and interaction errors, for each of the three participants. The observed autonomic response pattern to interaction errors, namely cardiac deceleration and increased phasic electrodermal activity conformed to findings from relevant literature [20,22,31,32]. Only Jason’s response to false negative errors deviated from this pattern. The average increase in phasic electrodermal activity was greater in response to false positives than to false negatives. This difference could be the effect of audio feedback following false positive switch activations versus no feedback stimuli of any kind following false negative instances. However, it would be plausible that false positive instances were actually more bothersome to the participants, because a false positive switch activation caused an unwanted change of state in the software.

Concerning the autonomic responses to true positive, the mild increases in phasic electrodermal activity found in Charles and Eric were consistent with the findings of Hajcak, McDonald and Simons [20] and Crone et al. [31]. Moreover, the weaker electrodermal response to true positive, a type of correct outcome, compared to interaction errors could be corroborated by these two studies. In terms of cardiac response, Hajcak, McDonald and Simons reported that cardiac acceleration accompanied correct outcomes. In contrast, Somsen et al. [49] reported cardiac deceleration with positive feedback but of lesser magnitude than the deceleration following negative feedback. The experimental tasks of the two studies differed (Stroop test versus Wisconsin Card Sorting), but both were nevertheless stimulus–response tasks in which the subject responded to each stimulus with a physical action. At a first glance, these findings seem inconsistent. However, Somsen et al. [49] remarked that enhanced cardiac deceleration following negative feedback is the consequence of delayed heart rate recovery due to error-related cognitive processing. Further, the cardiac deceleration and acceleration associated with positive feedback is actually part of the same heart rate recovery process. More importantly, the key difference between the cardiac responses to positive and negative feedback is greater deceleration in the latter case due to the heart rate recovery lag. Let us apply this knowledge to the cardiac responses observed in this study between true positives and false negatives, outcomes that involved physical responses (switch activation attempts) to stimuli. Table 2 indeed shows greater cardiac deceleration to false negatives for Charles and Eric, respectively, corroborating the findings of the aforementioned studies.

All three participants had inert autonomic response patterns to true negatives that were distinct from their response patterns to true positives and interaction errors. True positives and false negatives involved actions by the switch users, which would affect their physical and cognitive states. False positives were mostly unexpected by the switch users and thus could also modulate their cognitive and affective states. On the other hand, true negatives were not unexpected and did not require the switch user to act. The observed inert response patterns confirmed this supposed physical and physiological indifference to true negative events.

Charles and Eric had comparable responses to the events of single-switch activity. The few differences in response pattern and magnitudes could be attributed to individual response stereotypy [50]. Jason’s autonomic response patterns were obviously different from those of the other two participants. Differences in basal physical state between Jason and Charles or Eric during single-switch activity likely resulted in different autonomic response patterns. Charles’ and Eric’s relaxed body tone would be more congruent with the physical state of the able-bodied participants described in other studies from literature. Jason’s increased body tone, which was akin to that typically observed during light exercise, might have influenced his basal autonomic nervous system activity to the point of modifying his response pattern to false negatives. Thermoregulatory sweating might have been a contributing factor in distorting the electrodermal response patterns, such as the greater magnitude of phasic electrodermal response following true positives than false negatives, as well as the shift towards tonic electrodermal level as being the labile skin conductance component to false negatives. Finally, Jason’s struggles to relax his muscles during single-switch activity highlighted the individual motoric manifestations of cerebral palsy despite a common Gross Motor Function Classification Scale [34] level across participants.

Jason’s stronger phasic electrodermal response to true positives rather than to interaction errors may have also, in part, been attributed to a psychology of helplessness. While all three participants strived for high task accuracy, Jason insisted on knowing his score immediately after each activity block. Table 1 shows that Jason had seen relatively fewer true positives but more interaction errors than the other participants. Thus, Jason likely had less confidence and tended to underestimate his actual performance with the vocal cord vibration switch [16]. In this case, true positive events might have be more significant to Jason, resulting in enhanced phasic electrodermal response, because every true positive instance affirmed his skills with the switch. Incidentally, Jason’s phasic electrodermal response to false positives was actually comparable in magnitude to those of Charles and Eric, suggesting that false positive events were no less significant to Jason.

### Visual presentation of single-switch scanning

The visual presentation of single-switch scanning by the letter matching software deviated from conventional presentations, where the choices are fixed and the selectable item indicator traverses each choice. However, the variant visual presentation allowed the software to render the letters at a fixed size and large enough for the participant to view, even if the target letter was more than five choices away. It also removed the need to track a moving indicator. The participants should not have had difficulty coping with the change because the fundamental mechanisms of single-switch scanning remained intact.

### Limitations

The present case series featured a much smaller participant sample size than the other studies from the psychophysiology literature. This was because of difficulties with finding child single-switch users who were of comparable skill level and willing to commit to a long term study. Thus, the present case series can only conclude the existence, but not the generalization, of the specific autonomic response patterns to correct outcomes and interaction errors in individuals with severe cerebral palsy. In fact, the present case series revealed individual-specific autonomic responses to single-switch activity events, suggesting that further research with many more children with severe disabilities is required to ascertain the reality of arriving at a small number of generalized responses.

The present access pathway involved humming, a task which requires balanced control of the respiratory muscles to control the expiration of air [51]. As a consequence, the respiratory correlates of desired switch outcomes and interaction errors could not be quantified. To gauge respiratory responses associated with switch activity, future research will need to invoke access pathways that do not involve the airway.

Because the analysis was conducted from an ensemble approach, we cannot comment on the viability of detecting the distinct autonomic response patterns to correct outcomes and interaction errors on a single-trial basis. Nevertheless, evidence of specific autonomic responses to correct outcomes and interaction errors at an ensemble level is a prerequisite for eventual single-trial pattern recognition. We note that methods for single-trial recognition of the specific autonomic response patterns will have to be robust against movement artifacts, which are inevitable when measuring from individuals with severe cerebral palsy.

We advise against inferring the state of development of the anterior cingulate cortex of the three participants based on the reported autonomic response patterns. The association of the autonomic response patterns with activities of the anterior cingulate cortex in the cerebral palsy population need to be supported by analyses of functional brain image data, which was beyond the scope of the present analysis.

## Conclusions

The present case series investigated the autonomic changes of three pediatric single-switch users in response to correct outcomes and interaction errors during single-switch scanning. Each participant showed distinct autonomic responses to activity engagement and true positive, true negative, false positive and false negative events. The cardiac deceleration and increased phasic electrodermal activity following true positives and interaction errors were consistent with findings from the psychophysiology literature.

## Competing interests

The authors declare that they have no competing interests.

## Authors’ contributions

BL conceived the study, implemented data collection, conducted the physiological signal analyses and drafted the manuscript. TC provided guidance and supervision pertaining to the study and edited the manuscript. Both authors read and approved the final manuscript.
